# Segmented cell analyses to measure redox states of autofluorescent NAD(P)H, FAD & Trp in cancer cells by FLIM

**DOI:** 10.1038/s41598-017-18634-x

**Published:** 2018-01-08

**Authors:** Horst Wallrabe, Zdenek Svindrych, Shagufta R. Alam, Karsten H. Siller, Tianxiong Wang, David Kashatus, Song Hu, Ammasi Periasamy

**Affiliations:** 1 The W.M. Keck Center for Cellular Imaging, University of Virginia, Charlottesville, VA USA; 2 Departments of Biology, University of Virginia, Charlottesville, VA USA; 3 Biomedical Engineering, University of Virginia, Charlottesville, VA USA; 4 Advanced Research Computing Services, University of Virginia, Charlottesville, VA USA; 5 Microbiology, Immunology & Cancer Biology, University of Virginia, Charlottesville, VA USA

## Abstract

Multiphoton FLIM microscopy offers many opportunities to investigate processes in live cells, tissue and animal model systems. For redox measurements, FLIM data is mostly published by cell mean values and intensity-based redox ratios. Our method is based entirely on FLIM parameters generated by 3-detector time domain microscopy capturing autofluorescent signals of NAD(P)H, FAD and novel FLIM-FRET application of Tryptophan and NAD(P)H-a2%/FAD-a1% redox ratio. Furthermore, image data is analyzed in segmented cells thresholded by 2 × 2 pixel Regions of Interest (ROIs) to separate mitochondrial oxidative phosphorylation from cytosolic glycolysis in a prostate cancer cell line. Hundreds of data points allow demonstration of heterogeneity in response to intervention, identity of cell responders to treatment, creating thereby different sub-populations. Histograms and bar charts visualize differences between cells, analyzing whole cell versus mitochondrial morphology data, all based on discrete ROIs. This assay method allows to detect subtle differences in cellular and tissue responses, suggesting an advancement over means-based analyses.

## Introduction

Applications of Fluorescence Lifetime Imaging Microscopy (FLIM) have grown exponentially in a broad range of life-sciences and industrial fields, a reflection of specific advantages over intensity-based microscopy^1–5^. FLIM, when combined with FRET (Förster Resonance Energy Transfer), can establish the fraction of interacting and non-interacting donor fluorophores^6–13^. Importantly, fluorescence lifetime is independent of fluorophore concentration, which makes it a valuable tool for quantitative studies in scattering and absorbing samples. Both frequency domain and time domain FLIM methods have been applied^14–16^. This manuscript uses the latter, also called Time-Correlated Single Photon Counting (TCSPC)^17^. Multiphoton excitation conveniently excites molecules that would otherwise require excitation in the UV region, generally injurious to live cells at longer exposure.

Mitochondrial oxidative phosphorylation (OXPHOS) activity consumes NADH (increased NADH-enzyme-bound fraction) and produces FAD (diminished FAD enzyme-bound fraction). Both the co-enzymes in their reduced (NAD(P)H and FADH_2_) and oxidized (NAD(P)^+^ and FAD) forms participate in the cellular oxidation-reduction reactions critical for cell physiology. In cancer, a higher glycolytic rate is a less efficient way of producing energy (2Pyruvate + 2ATP + 2NADH) than the low glycolytic rate and mitochondrial oxidation of pyruvate (36 ATP) seen in normal cells^18^. The interplay between glycolysis and OXPHOS is changed in different cancers and involvement of other pathways like elevated mitochondrial glutaminolysis is also seen in prostate cancer (PCa).

The coenzymes NADH and FAD are involved in catabolic reactions of amino acid and fatty acid oxidation, glycolysis, citric acid cycle and in electron transport chain (ETC) which ultimately results in energy generation by oxidative phosphorylation (OXPHOS). NADPH is mainly involved in anabolic reactions, which use energy for biosynthesis. Previous reports have shown that Tryptophan (Trp) lifetime (as donor) is quenched through FRET interaction in the presence of NADH in solution^19–23^. We are introducing a novel hypothesis to analyze Trp–NAD(P)H interactions in the context of cellular metabolism. Several enzymes involved in NAD^+^/NADH conversion carry Trp residues and are potential candidates: Lactate dehydrogenase (6 R)^20^, Glyceraldehyde 3-phosphate dehydrogenase (3 R)^24^, Isocitrate dehydrogenase (8 R)^25^, Malate dehydrogenase (5 R)^20^, Glutamate dehydrogenase (5 R)^26^.

In cancer, there is metabolic re-programming and variable interaction between the glycolytic and OXPHOS energy generation. Cancer cells unlike normal cells often produce energy via glycolysis followed by the production of lactate even in presence of oxygen (Warburg Effect)^27^. Usually, cancer cells have glycolytic rates up to 200 times higher when compared to their respective normal tissue and some have defective OXPHOS activity as a strategy to interfere in the apoptotic pathways^28^. A higher glycolytic rate in cancer is a less efficient way of producing energy (2Pyruvate + 2ATP + 2NADH) than the low glycolytic rate and mitochondrial oxidation of pyruvate (36 ATP) seen in normal cells^18^. However, cancer cells shift their metabolism to the production of lactate from pyruvate in the cytosol by the enzyme Lactate dehydrogenase (LDH), in the process oxidizing the NADH and regenerating NAD+ required for ATP production through glycolysis.

Tracking the auto-fluorescent signals of the co-enzymes NAD(P)H and FAD in combination with an intensity-based FAD/NAD(P)H redox ratio has been well established by Chance *et al*. in 1979 as a basis to measure the overall redox state in cells since the FAD/NAD(P)H are near oxidation-reduction equilibrium, the ratio of the two fluorescence intensities, suitably normalized, approximates the *in vivo* oxidation-reduction which offers a foundation for the resolution of the Redox states in 2- and 3- dimensions, which we have investigated in this manuscript using FLIM. Mitochondria, the power house of a cell has prominent and discrete signals from NAD(P)H and FAD and provides a “consumer report” of energy expenditure and generation, its redox state and the level of metabolic activity^29^.

Genetically encoded fluorescent redox sensors^30^ offer alternative approaches to investigate cellular metabolic states in a variety of specimen types, particularly in cancer applications. Unfortunately, light scattering and absorption - especially in tissue specimens - makes intensity-based methods problematic or unusable.

This paper’s main emphasis is on exploring FLIM microscopy’s potential for greater depth analyses of the metabolic states of cancer cells. We expanded the common FLIM assay parameters by introducing a novel Fluorescence Lifetime Redox Ratio (FLIRR) measurement, NAD(P)H-a2%/FAD-a1%. We also propose an additional marker for metabolic changes by a Trp-NAD(P)H FRET assay. From an imaging point of view this is made possible as both, Trp (3-photon) and NAD(P)H (2-photon), are excited simultaneously by the 740 nm laser.

We assessed the metabolic response under three different interventions in cancer cell monolayers as proof-of-principle to show the applicability of the assay: African-American (AA) prostate cancer cells (PCa) were (a) starved overnight in HBSS with 5.5 mM glucose, to slow down their metabolic activity followed by stimulation with 25 mM glucose for 30 min, in their normal growth media. HBSS starving still provides 5.5 mM glucose, but no growth factors and maintains cells in a reduced metabolic state. (b) 50 µM CoCl_2_ treatment overnight followed by 25 mM glucose growth media for 30 min. CoCl_2_ is a hypoxia mimetic agent that targets the Complex I enzyme preventing the conversion of NAD(P)H to NAD^+^ in the electron transfer chain (ETC) of the mitochondria and affecting the cellular respiration; as the effect wears off and glucose is added the cells are expected to recover to their individual redox states (c) treatment with 1 µM doxorubicin for a 60 minute time course^28^. Doxorubicin is a potent anti-cancer drug that affects cancer cell DNA (both nuclear and mitochondrial) interfering with replication and by doing so restoring the otherwise impaired apoptosis pathway, regulated by the mitochondria. In all cases, controls and treatment have identical fields-of-view (FoV).

## Results

### Cell segmentation and FLIM data analysis in discrete thresholded ROIs

Lifetime measurements of NAD(P)H, FAD and Trp are analyzed by our FLIM assay for quantitative analysis of differential lifetimes, their relative fractions as ratios, such as enzyme-bound/unbound NAD(P)H, and energy transfer efficiencies (E%) between Trp and NAD(P)H^31,32^. As NADH and NADPH cannot be readily separated based on their spectral properties, it is conventionally written as NAD(P)H; we have also chosen to use NAD(P)H to represent both.

Calculation of mean values of individual image data or complete cell data within an image may be sufficient to meet some objectives but may not extract discrete inter-cell heterogeneity, particularly in response to interventions, as cell responses vary. Our FoV-based assay method acquires images pre- and post-intervention and analyzes individual cell responses within the same image to capture heterogeneities and to track differential responses; a multi-parametric approach also includes correlation with ROS and apoptotic markers^28^ (not presented here). Our quantitative analysis method of FLIM images allows cell segmentation within a FoV and is based on 2 × 2 pixel regions of interest (ROI) in cells within each image (FoV)^10,11,33^ to isolate *inter alia* sub-populations. This assay method detects subtle differences in cellular responses in these heterogeneous sub-populations, suggesting an advancement over means-based analyses.

**Figure 1:**
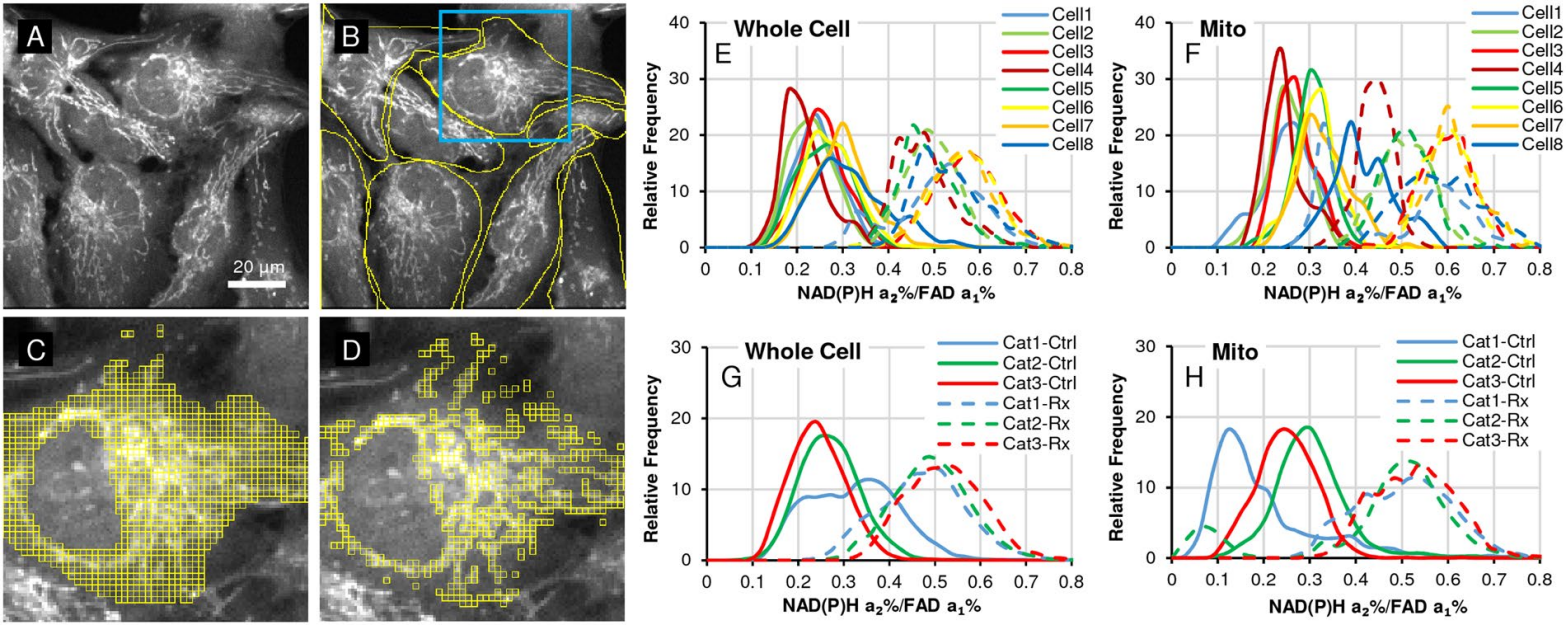
Image analysis sequence. After FLIM data fitting in SPCImage and data export, (**A**) NAD(P)H photon images are used to match morphology with thresholded ROI selection. (**B**) Individual cells are segmented in the field-of-view (FoV) (currently manually). (**C**) 2 × 2 pixel ROIs are automatically generated for the whole cell and (**D**) differentially thresholded to capture the more concentrated NAD(P)H signals of the mitochondrial morphology^28^. Then, ImageJ custom macro allocates 2 × 2 pixel ROI data to segmented cells (see **B**). (**E** & **F**) Microsoft EXCEL macros generate *inter alia* frequency distribution histograms by segmented cells; the example shown here is for the FLIM redox ratio of whole-cell and mitochondrial data. (**G** & **H)** Based on each cell’s response to treatment by median change from control to treatment, cells from all FoVs are classified as low (Category1), medium (Category2) and high responders (Category3) and merged in these 3 categories. Subsequent figures - where applicable – were analyzed the same way only showing the merged data by category.

Following the cellular morphology, 2 × 2 pixel ROIs are automatically generated based on photon counts in the FLIM NAD(P)H emission channel, and by setting lower and upper thresholds in a custom ImageJ plugin, described in the Supplementary Information. The thresholds are flexibly adjusted to isolate mitochondrial regions and secondly, the complete cell area (without the nucleus, which is zeroed before ROI selection) (Fig. 1). We chose NAD(P)H photon counts, which best matched simultaneously imaged MitoTracker (see ref.^28^/Figure S1). For robust analyses, a large number of ROIs are generated to capture inter-cell differences by segmented cell information. Cell segmentation by FoV is currently manually executed in ImageJ (see Supplementary Information).

The number of data parameters for each ROI from 3 FLIM detectors for 3 auto-fluorescent probes excited at different wavelengths and emitting in different spectral ranges produce large image data sets. This paper presents a modular and adjustable method to process these large FLIM image datasets, combining ImageJ (NIH) and Excel (Microsoft Office) with custom macros and template spreadsheets. Merged cell data from several different FoVs compares whole-cell with mitochondrial data sets and establishes distribution frequencies and correlations for each of the parameters (Fig. 1). The key feature of the ROI approach generating multiple data points – instead of one mean value per cell – is the ability to highlight inter-cell variabilities, made more robust by re-imaging the same FoV.

Comparing segmented whole-cell (without nucleus) data with mitochondrial morphology-based data, both generated by 2 × 2 pixel ROIs, allows to deduce contributions from OXPHOs and glycolysis to the cellular redox states and quantifies the heterogeneous nature of cell responses. NAD(P)H signals come from the mitochondrial OXPHOS and cytosolic glycolysis, while FAD signals originate from the mitochondria only, all captured in whole-cell analyses for a total cell (without nucleus) redox assessment. Isolating mitochondrial morphology by the same FLIRR assay quantifies the contribution from OXPHOS, allowing the difference to whole-cell data to be attributed to glycolysis. Our data imply that changes in FLIRR in mitochondrial OXPHOS are driven by increasing enzyme-bound NAD(P)H- a2% and decreasing enzyme-bound FAD-a1%, while changes in cytosolic glycolysis can be attributed solely to changes in the enzyme-bound NAD(P)H a2% fraction in the absence of FAD.

After having demonstrated the heterogeneity of cell response to treatment (Rx) by individual cell, we classify all cells from different FoVs into 3 categories, based on their percent median change of FLIM redox ratio (described below). This reduces the data complexity and visualizes the trends of each category, identity of cell responders to treatment, creating thereby different sub-populations (Fig. 1). In different situations, 2 or 4 categories may be just as appropriate for different experimental objectives.

### Novel FLIM based redox ratio (FLIRR) validates response to treatment under different conditions

As touched on in the introduction, the major energy generating metabolic pathways in the cell are glycolysis, citric acid cycle and oxidative phosphorylation (OXPHOS). Mitochondria, the power house of the cell accounts for the 95% of energy generation in the form of ATP through the OXPHOS cycle. Glycolysis and other catabolic pathways feed in their substrate/end products and electron carriers (NADH/FADH_2_) into the citric acid cycle, finally entering into the OXPHOS cycle. Taken together, OXPHOS cycle is the main stage for the generation of energy in the form of ATP by FoF1 ATPase^34^.

Therefore, the mitochondrial redox pair FAD - NADH provides information on the redox state of the cell. During the OXPHOS cycle NADH gets converted to NAD^+^ by the enzyme NADH dehydrogenase, for this to happen NADH has to be in an enzyme bound state (more NADH-enzyme-bound fraction-a_2_%); whereas, FADH_2_ gets converted to non-enzyme bound FAD by the enzyme succinate dehydrogenase (less FAD enzyme-bound fraction-a_1_%)^34^. The *a*_1_ and *a*_2_ are the pre-exponential parameters associated with the shorter (*τ*_1_) and longer (*τ*_2_) lifetime components of a bi-exponential fluorescence decay model. These parameters are determined by fitting the model to the measured fluorescence decay data on a per pixel basis. *a*_1_% and *a*_2_% are normalized parameters according to *a*_1_% = *a*_1_/(*a*_1_ + *a*_2_) and *a*_2_% = *a*_2_/(*a*_1_ + *a*_2_), thus removing the effect of the overall fluorescence intensity on the parameters.

**Figure 2:**
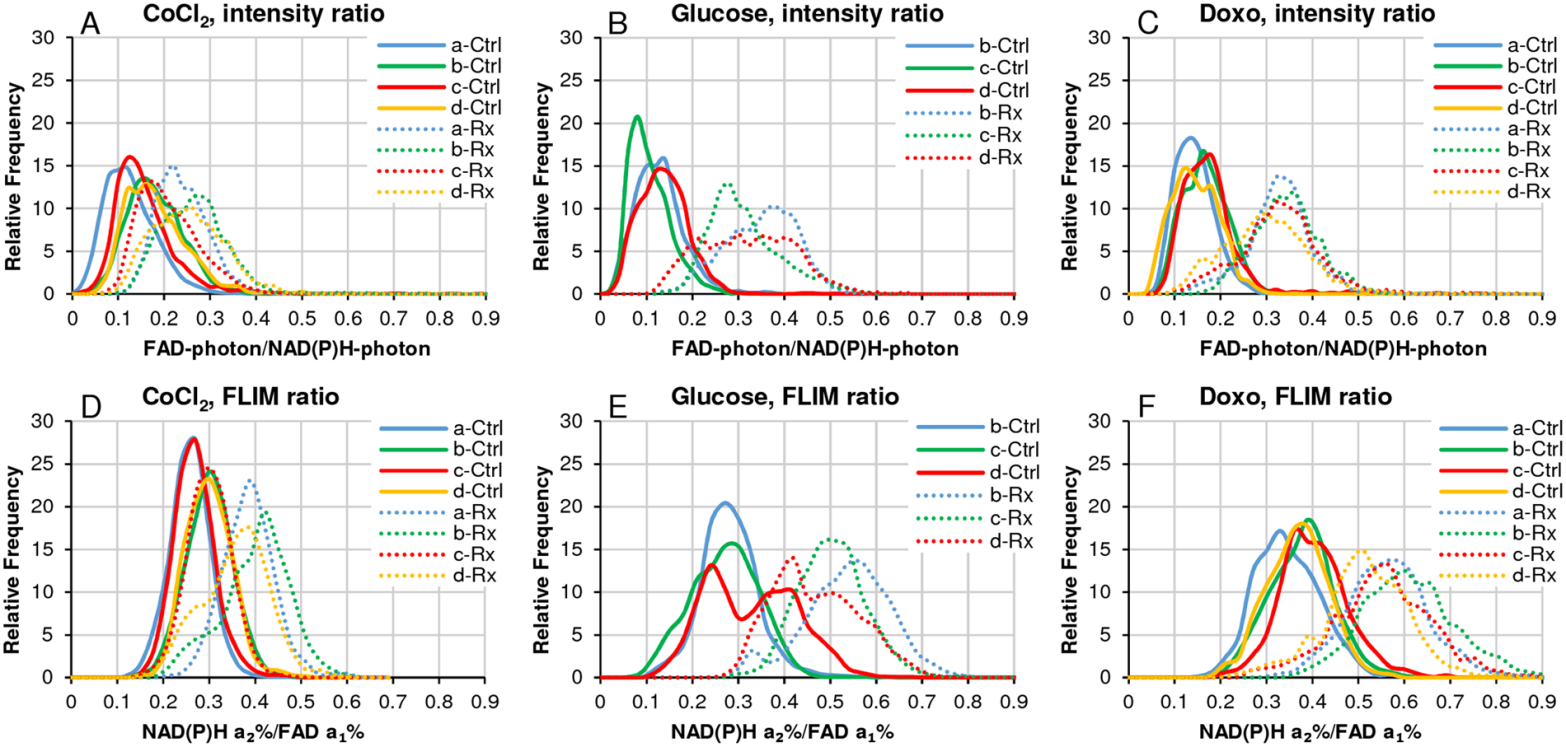
Comparison between intensity ratios and FLIRR for redox states for each of the 3 experimental groups. (**A–C**) Charts display the common intensity-based redox ratios FAD/NAD(P)H for the 3 experimental conditions explored in this manuscript. **(D–F)** Charts display the suggested alternative FLIRR -NAD(P)H-a2%/FAD-a1% - the ratios of the enzyme-bound fractions of each co-enzyme. See text for further details.

Using FLIRR was conceived for live tissue specimen applications to overcome the intensity-based light-scattering and absorption barriers in thicker specimens. In the commonly used intensity-based FAD/NADH ratio^29,35^ increased metabolic activity is denoted by increases in the ratio, due to the conversion of fluorescent NADH to non-fluorescent NAD^+^ and conversion of non-fluorescent FADH_2_ to fluorescent FAD. FLIRR - also increases, meeting the same redox parameter objective, unaffected by intensity-related limitations. We validated FLIRR against the intensity ratio for consistency, possible in monolayer cell cultures, as proof-of-principle under the 3 different interventions (Fig. 2).

### Heterogeneity in redox ratio response under starvation and upon glucose challenge in PCa cells

**Figure 3:**
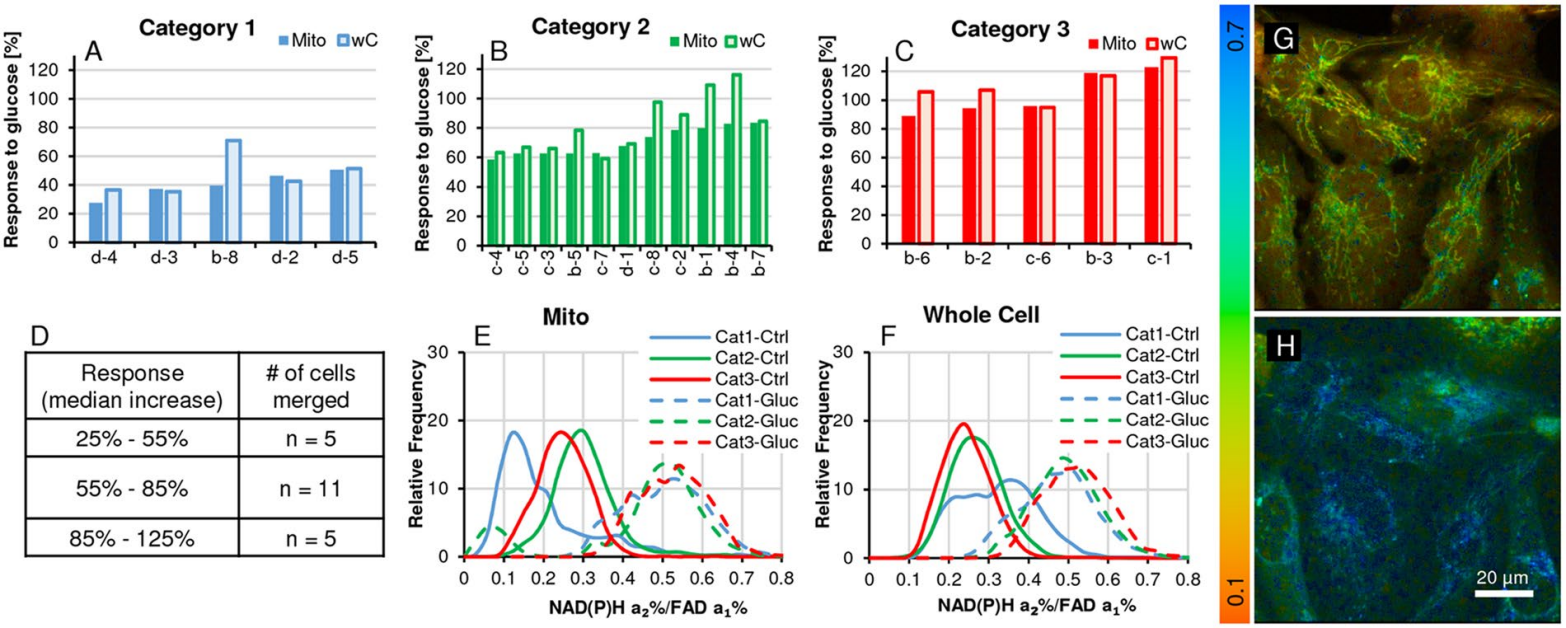
Heterogeneity in redox ratio response under starvation (5.5 mM glucose, control) and upon glucose challenge (25 mM glucose/30 min) in AA PCa cells. Segmented cells were analyzed as described in Fig. 1 as individual cell histograms. (**A**–**C**) The X-axis denotes segmented cell identifiers, the Y-axis the % median increase of FLIRR from control to glucose challenge in the 3 responder categories (low, medium, high). The increases of the median ratio of mitochondrial (‘mito’) vs. whole-cell (‘wC’, without nucleus) in the same cell are compared; we are suggesting that where the increase is comparable, metabolism is driven by mitochondrial OXPHOS and where wC data exceeds mitochondrial ROIs, the incremental rise can be attributed to glycolysis as in cells d-4, b-8 in chart A and b-5, c-8, c-2 etc. in chart (**B**). (**D**) Ranges of % median changes of FLIRR based on mitochondrial data with the number (n) of cells in each category which are merged together. (**E**,**F**) Merged FLIRR frequency distribution of the 3 response categories of starved controls vs. glucose challenge. (**G**,**H**) Representative FLIRR images before and after the glucose challenge, showing the rise in FLIM redox ratio corresponding to increase in metabolic activity.

In Fig. 3, the percent change of the median value - from control to glucose challenge - within each segmented cell is first calculated, using the mitochondrial data as the basis, provides a range of responses to the glucose challenge. The cell data from n = 21 cells is allocated to 3 response categories: 25–55%, 55–85% and 85–125% (see 3D). In 3A–C, median values of mitochondria vs whole cell are compared in these 3 categories. 3E & F show the distribution frequency of ROI data points by category for control vs glucose challenge and comparing mitochondria with whole cell data respectively. There is more heterogeneity in the control cell sub-populations in mitochondrial ROIs as compared to whole cell data. We suggest that the difference is seen due to the greater impact of starvation on glycolysis which identifies differential ongoing defective OXPHOS in these cohorts. Those differences are also apparent in the bar charts. Addition of glucose restores normalcy as seen in both E & F. In 3 G & H, representative color-coded images visualize FLIRR for control and glucose challenge with a shift to a higher ratio meaning a higher metabolic activity.

### Heterogeneity of FLIRR response to CoCl_2_-treatment and upon glucose challenge in PCa cells

The CoCl_2_ pre-treatment and glucose challenge data follows the same pattern as the ‘starvation’ experiment (Fig. 3), but affecting cells pharmacologically differently. Shutting down the ETC impacts mitochondrial signals and the comparison of mitochondrial and whole cell ROIs is somewhat different; it has to be kept in mind that NADH & FAD (FAD exclusively) signals are concentrated in the mitochondria. In 4A–C, median response of mitochondria vs whole cell are compared in these 3 categories. 4E & F show the distribution frequency of ROI data points by category for control vs glucose challenge and comparing mitochondria with whole cell data. We are suggesting that where the increase is comparable, metabolism is driven by mitochondrial OXPHOS and where whole cell data exceeds mitochondrial ROIs, the incremental rise can be attributed to glycolysis. As mentioned under the previous heading, CoCl_2_ controls (unlike starved) are more closely matched, but glucose treatment results in different recovery patterns, made more visible when merged into sub-population response categories. Interestingly, starved segmented cell controls show greater variability than CoCl_2_ pretreated controls, presumably based on the particular energy needs of some cells adjusting better than others to the HBSS lower glucose availability. In contrast, CoCl_2_ a hypoxia mimetic agent inhibits complex I and is more likely to shut down the ETC^36,37^ affecting cells more evenly. Representative color-coded FLIRR images are shown in 4 G &H – pre-treated CoCl_2_ control and after 30 min glucose challenge. A shift to a higher FLIM redox ratio shows higher metabolic activity.

### Heterogeneity FLIRR response upon treatment with anti-cancer drug doxorubicin in PCa cells

**Figure 4:**
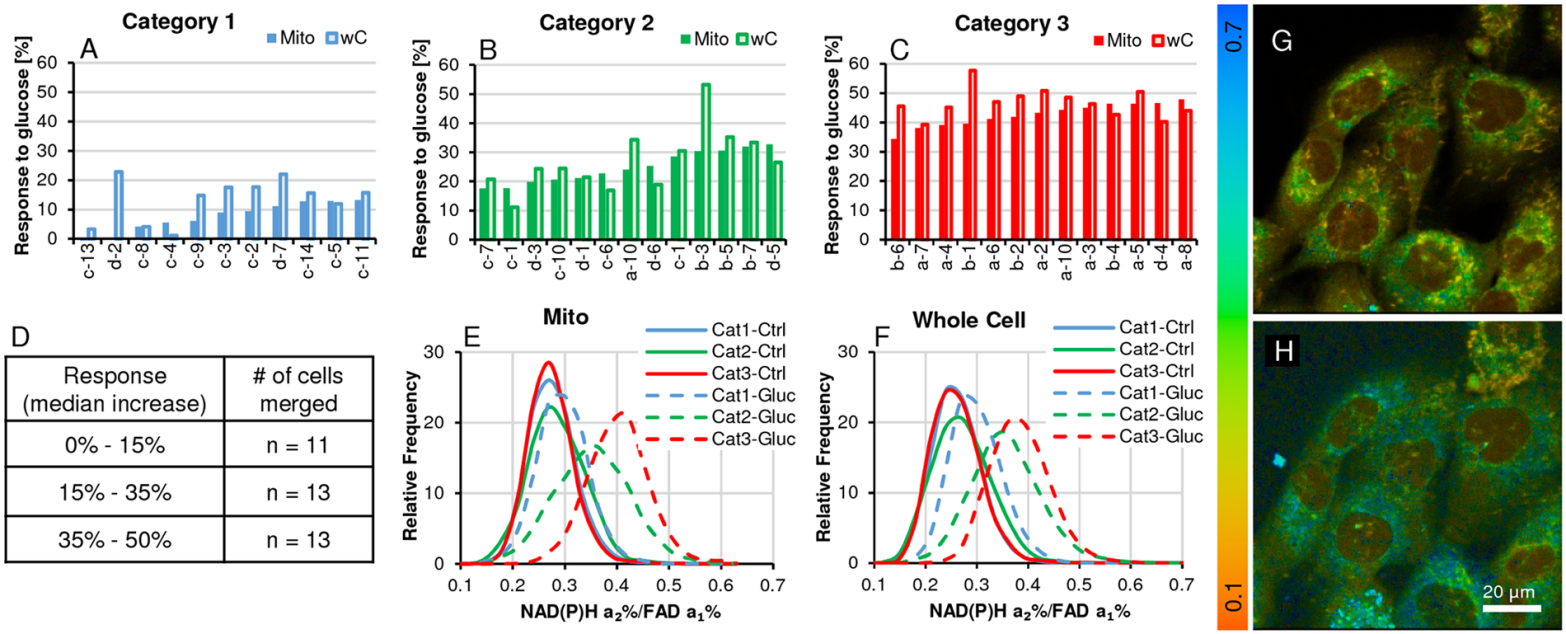
Heterogeneity of FLIRR response with 50 µM CoCl_2_-treated overnight controls and upon glucose challenge (25 mM glucose/30 min) in AA PCa cells. Segmented cells were analyzed as described in Fig. 1. (**A**–**C**) The X-axis denotes segmented cell identifiers, the Y-axis the % median increase of FLIRR (NAD(P)H-a2%/FAD-a1%) from CoCl_2_ treated control to glucose challenge in the 3 responder categories (low, medium, high). The increases of the median ratio of mitochondrial (‘mito’) vs. whole-cell (‘wC’, without nucleus) in the same cell are compared; we are suggesting that where the increase is comparable, metabolism is driven by mitochondrial OXPHOS and where whole cell data exceeds mito, the incremental rise can be attributed to glycolysis as in cells d-2, c-9 etc. in chart A and d-3, a-10, b-3 etc. in chart (**B**). (**D**) Ranges of % median changes of FLIRR based on mitochondrial data with the number (n) of cells in each category which are merged together. (**E**,**F**) Merged FLIRR frequency distribution of the 3 response categories of CoCl_2_ treated controls vs. glucose challenge. (**G**,**H**) Representative FLIRR images before and after the glucose challenge, showing the rise in FLIM redox ratio.

To extend the assay to an application of anti-cancer drug treatment example, identical FoVs were acquired for AA PCa cells, untreated controls and time points (15, 30, 45, 60 min) after treatment with doxorubicin. Individual cell segmentation and analysis plus categorization was carried out as described in Figs 3 and 4. The median response to doxorubicin is calculated against control for each time point. In view of the number of individual cell data points (n = 21, ROI data points Ctrl = 25,000), bar charts are not shown. Again, frequency distribution of mitochondrial data (5A_C) is compared with whole-cell data (5D-F) within response categories. 5 G & H summarizes the differential response in terms of the percent change of the median FLIRR by time point, comparing mitochondrial- with whole-cell data. Representative time-series images of the increasing FLIRR after treatment (5I) shows increase in OXPHOS activity before the on-set of apoptosis^28^.

### Trp-E% vs. FLIRR correlation analysis offers an additional method to track metabolic changes

The rationale for tracking Trp has been described in the introduction, allowing to calculate energy transfer efficiencies (E%) between Trp (donor) and NAD(P)H (acceptor) as a classical FRET event. Different enzymes in the glycolysis and mitochondrial OXPHOS carry Trp residues, which are candidates for this interaction and offering an additional marker of metabolic changes and potentially an approach to differentiate glycolysis from oxidative phosphorylation – particularly relevant to cancer cells.

To demonstrate the relevance of this FRET assay, we have analyzed the above doxorubicin time-course treatment in AA PCa cells. To be consistent with the categorization applied in Fig. 5, E% data of whole cell and mitochondrial ROIs were segregated into the identical 3 categories of low, medium and high median response as per their FLIRR-based value.

**Figure 5:**
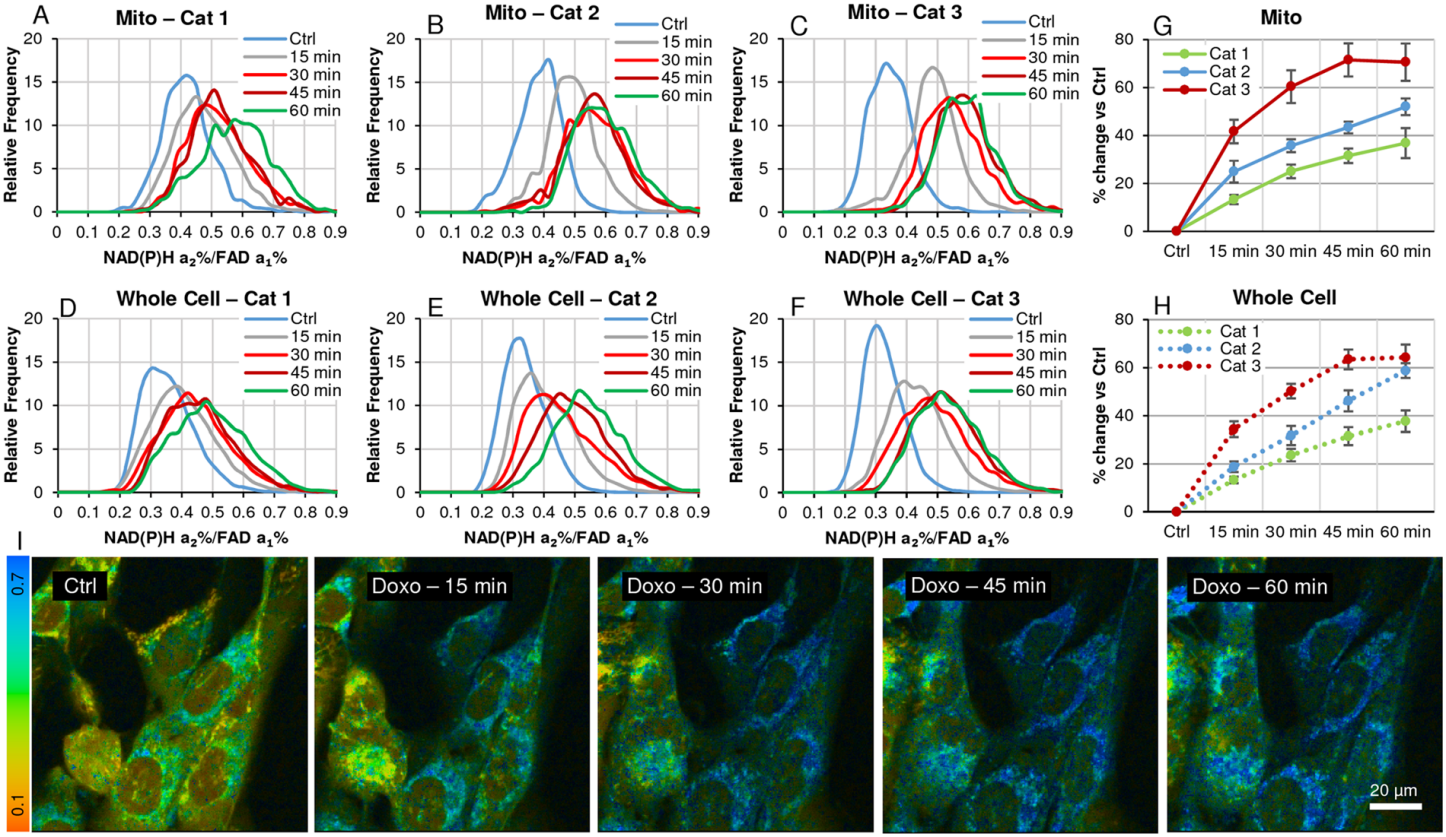
Heterogeneity FLIRR response upon treatment with anti-cancer drug doxorubicin in AA PCa (Control, 15, 30, 45, 60 min). Segmented cells were analyzed as described in Fig. 1. To reduce the complexity, cell data was sorted by their response to treatment (low, medium, high), measured by FLIRR, then merged within the three responder categories. (**A–C)** Responder categories by time point for mitochondrial morphology data, **(D–F)** responder categories by time point for whole-cell (without nucleus) data. **(G**,**H)** Median response vs. untreated control by time point for each of the responder categories. **(I**) Images represent the continuing effects of doxorubicin on increasing FLIRR before the onset of apoptosis^28^.

**Figure 6:**
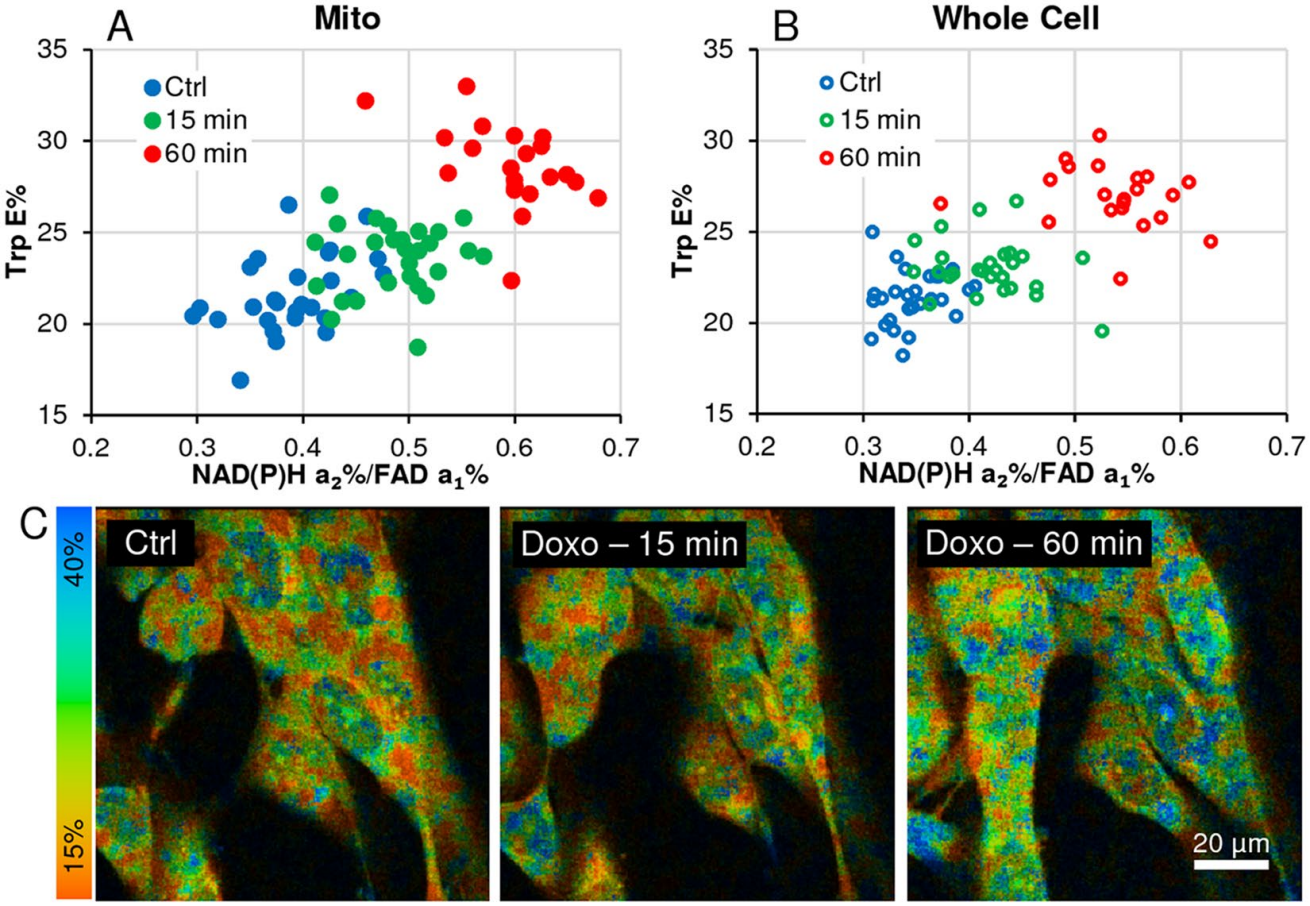
Trp E% vs. FLIRR correlation analysis offers an additional method to track metabolic changes. (**A**,**B**) Correlation of FLIRR vs. Trp E% by segmented cells for Control and 3 doxorubicin treatment time points (Ctrl ->15 and 60 min), for both, mitochondrial and whole-cell morphology analyzed by 2 × 2 pixel ROI data points (nuclear regions are not included in the analysis) shows Trp E% and FLIRR correlation, tracking metabolic response to treatment in cancer cells. (**C**) Color coded images show increase in E% with treatment. Correlation coefficients, control to 60 minute treatment: (**A**) r = 0.70; (**B**) r = 0.69.

Median FLIRR data of n = 21 cells is correlated with E% (Fig. 6A,B) for control, 15 min and 60 min doxorubicin treatment, comparing mitochondrial with whole cell, showing a clear interdependency between the two parameters; E% rises with increasing redox states as measured FLIRR.

### Application of FLIRR *in vivo* in a tumor xenograft mouse model

**Figure 7:**
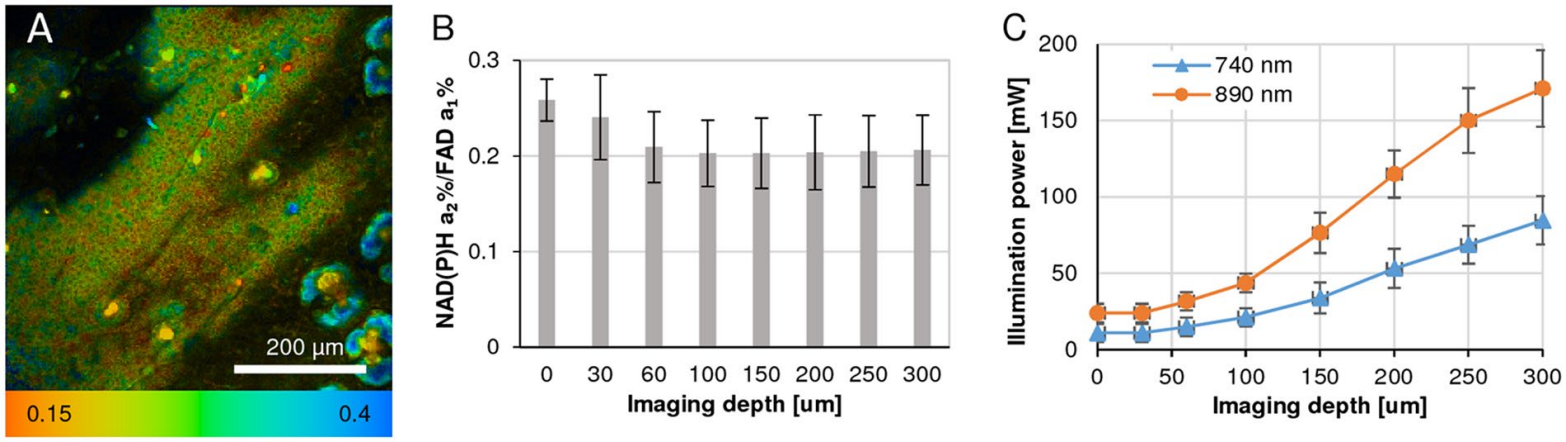
Application of FLIRR *in vivo* in a tumor xenograft mouse model. (**A**) Image of NAD(P)H-a_2_%/FAD-a_1_% FLIRR, 60 um below skin surface (25 × 0.8NA immersion lens). (**B**) Mean values of FLIRR at different imaging depths, based on the same 2 × 2 pixel ROI assay applied in the AA PCa cell monolayer experiments. ROI selections are made based on the intensity of NAD(P)H and FAD fluorescence, avoiding bright features (e.g. sebaceous glands). At depths of 0 and 30 µm the fluorescence originates predominantly from the epidermis and FLIRR deviates from that of the tumor. (**C**) The excitation power levels were increased with increasing imaging depth to account for light scattering and absorption. The overall fluorescence intensity was kept close to 10^5^ photons/s to avoid saturation of the FLIM detection system.

As mentioned earlier, the motivation to develop an alternative FLIM-based redox measurement arose from our work with live murine xenografts, where intensity-based measurements are unsuitable because of wavelength- and depth-dependent light scattering and absorption. In a mouse cancer xenograft model, the tumor was imaged at different depths collecting NADH and FAD images at 740 nm and 890 nm illumination, respectively (Fig. 7A). FLIRR values are consistent across all depths within the tumor (Fig. 7B), even though the excitation light intensity was increased significantly with increasing imaging depth (Fig. 7C). The combined effects of light scattering, absorption and optical aberrations of the excitation laser light cause rapid decrease of the peak illumination intensity in the focus with increasing imaging depth within an inhomogeneous tissue. While these effect can be partially counteracted by increasing the excitation power, it is impossible to maintain constant two-photon excitation intensity at different depths and two different wavelengths, a requirement for measuring intensity-based redox ratio deep in tissue, which makes the application of a 2-photon intensity-based redox measurement unachievable.

## Discussion

This method paper describes a number of novel approaches to image analysis involved in cell metabolic processes, utilizing the auto-fluorescent properties of NAD(P)H, FAD and Trp, captured by fluorescence lifetime microscopy (FLIM). While this technique is well established, combined with a commonly used intensity-based redox measurement (FAD/NAD(P)H photon ratio), this ratio is unsuitable for tissue sections, xenografts and the like, because of light-scattering and the need for varying laser power levels to acquire sufficient photons. Besides, having collected additional information only FLIM can provide vs. intensity parameters, we sought to propose an alternative FLIM-based redox measurement, NADH-a_2_%/FAD-a_1_% ratio (FLIRR), which we have validated against the standard intensity ratio (Fig. 2). In the course of oxidative phosphorylation, NAD(P)H-enzyme-bound fraction-a_2_% increases and FAD enzyme-bound fraction-a_1_% decreases, leading to a change in FLIRR. This FLIRR assay was applied to the 3 different interventions in AA PCa cells monolayer experiments (Figs 3–5) as proof-of-principle and to the xenograft experiment (Fig. 7), where the intensity redox measurement was unusable.

Our 3-detector FLIM board allowed us to capture simultaneously signals of auto-fluorescent Trp and NAD(P)H at 740 nm excitation, followed by FAD signal acquisition at 890 nm (Supplementary Information). Trp at 2-component FLIM fitting demonstrates the two moieties of unquenched higher lifetimes and quenched lower lifetimes as a result of FRET interactions between Trp (donor) and NAD(P)H (acceptor). These FRET events have been reported with molecules in solution^19–23^. Since several NAD(P)H interacting enzymes carry Trp residues, we hypothesized that increased metabolic activities should lead to more quenched Trp and subsequent increase of energy transfer efficiencies (E%), as the levels of enzyme-bound NAD(P)H increase. This indeed was shown in Fig. 6, correlating FLIRR with E%. While open questions remain, which Trp residues of which enzymes interact with NAD(P)H under which precise circumstances, we are suggesting that this novel marker could expand the possibilities of measuring redox states in different cancer models.

Re-imaging the same FoV – where possible – has the important benefit of being able to track specific cell populations based on their metabolic profiles. Where longer treatment times make application on stage impractical, future work could be extended to specimens in glass-bottom dishes with fiduciary markers. Controls could be imaged, coordinates recorded with respect to the fiduciary marker, treatment applied, dishes returned to the incubator and re-imaged for the same FoVs after 12, 24 hours or longer.

Heterogeneity of live specimens in their natural state and response to experimental interventions is well established. At quantitative analysis of image data, mean values of FoVs or cells within FoVs may be quite sufficient to arrive at some experimental conclusions, but may also miss subtle differences, providing further insights by displaying gradients and distribution frequencies by cell. We applied in this manuscript two extra steps: to generate data points based on 2 × 2 pixel ROIs, which select for morphological features by thresholding intensity to isolate mitochondria and whole cell (without nucleus). This provides a basis to compare two cellular morphologies and infer the interrelated OXPHOS vs. glycolysis dynamics. In Figs 3 and 4(A–C) bar charts compare the % increase of median FLIRR of mitochondria with whole cell (which includes the mitochondrial data) by cell. The incremental increase in the whole-cell bar is attributed to glycolysis; where there is no difference, glycolysis has not responded to the addition of glucose. This response difference can of course be presented in a number of different ways (e.g. normalization vs. control).

Comparing segmented cell data by whole-cell and mitochondrial morphology is an important feature of the assay allowing to deduce metabolic contributions from glycolysis vs OXPHOS, respectively. Accepted practices in the field base the intensity redox ratio on whole cells (without contributions from the nucleus). This manuscript extends the analysis by FLIRR and a 2 × 2 pixel ROI assay, including a whole-cell (without nucleus) data collection. Both, FLIRR and intensity ratio ignore the fact that NAD(P)H signals come from the mitochondrial OXPHOS and cytosolic glycolysis, while FAD signals originate from the mitochondria only, yet for a global whole-cell redox assessment an acceptable and practical metric. Our assay was further extended to isolate mitochondrial morphology by the same FLIRR assay to visualize the contribution from OXPHOS, suggesting that the difference between this and whole-cell data has to be attributed to glycolysis; we believe this to be an advancement over the established analysis methods, highlighting and quantitating the heterogeneous nature of cell responses, based on this difference (Figs 3–5).

Secondly, having isolated the two morphologies in two separate steps, cells in each FoV are segmented, generated ROIs are allocated to those cells. Histograms for each cell are automatically produced by custom macros (not shown), and allocated to 3 response categories for a less complicated interpretation of the results, based on their median response to interventions. Allocating segmented cells to 3 response categories may appear arbitrary, but is based on the data falling into specific ranges, which also resulted in comparable number of data points in each range. It is interesting to note that by starving cells, the controls show varied responses to starvation, which were leveled by the glucose challenge to normalcy. In contrast, CoCl_2_ reduced controls to similar levels, but upon 30 minutes glucose addition, cells recovered at different rates, showing heterogeneity in response. The third experiment of doxorubicin treatment time course – while analyzed in a monolayer of cells – is closest to conditions encountered at clinical conditions; experimentally, it can be flexibly applied in similar ways to organoids and tissue sections, depending on the experimental objectives and conditions. It would be ideal to have a normal/healthy redox value, which potentially could be approximated by using a Normal Human Prostate Epithelial (NHPEs) primary cells – something to be considered in the future.

Cancer cell’s known heterogeneity within tumors, determining the level of aggressiveness, their varying dependence on glycolysis and/or oxidative phosphorylation and other metabolism-related parameters make these pathologies prime targets to investigate heterogeneous populations.

With the assays presented here, existing image data can be further analyzed to discover sub-populations, differential responses to interventions causing cell heterogeneity and other sources for different cell behavior. For example, known *in-vitro* knock-down efficiencies can be correlated to individual cell image data and non-responders legitimately excluded from the analysis or separately analyzed. Specific cellular features are isolated by ROI thresholding; in the event that gray-level intensities or other parameters do not work well, specific cellular features can be manually extracted before applying ROI selection. The segmented analysis method presented here is flexible and applied to very specific FLIM auto-fluorescent targets, but is equally suitable for other FLIM image data.

## Materials and Methods

### Cell Culture

A PCa cell line E006AA from African-American origin has been used in this study. The E006AA (or AA) cells were maintained in high-glucose Dulbecco’s Modified Eagle Medium (Life Technologies) supplemented with 10% cosmic calf serum (Hyclone), 1% Penicillin-Streptomycin (Life Technologies), and 4 mM Sodium Pyruvate (Life Technologies). All cells were maintained in the cell culture incubator, at 37 °C with 5% CO_2_.

### Tumor xenograft mouse model

In Cancer xenograft mouse model HEK-Ras12v cell lines were injected into the ears of nude immune-compromised mouse. All mice handling and imaging experiments were done under the auspices of the University of Virginia Animal Care and Use Committee.

### Fluorescence Lifetime Imaging Microscopy and Different interventions in PCa cells

For imaging E006AA PCa cells were plated onto 25 mm round #1.5 glass coverslips (Thermo Scientific), in the growth medium and grown to 70–80% confluence. The cells were starved overnight in HBSS (Hank’s Balanced Salt Solution containing with 5.5 mM glucose) or treated overnight with 50 µM CoCl_2_ containing growth media. Untreated controls were included. Fluorescence lifetime imaging was acquired in phenol-free or in FluoroBrite-DMEM (Thermo Fisher Scientific) growth medium with the microscope-heated stage maintained at 37 °C and under the flow of humidified blood-gas mixture (5% CO_2_). After imaging of the control FoVs, either 25 mM glucose or 1 µM doxorubicin final concentration was added in the growth medium on stage and FLIM data was again acquired at 30 min post glucose challenge or at 15 min interval up to 60 min in-case of doxorubicin treatment.

### Fluorescence Lifetime Imaging Microscopy of cancer xenograft model

The mouse was positioned on a custom heated stage (Newport model 281 Lab Jack for coarse focusing, two Thorlabs MT1B Linear Stages for lateral positioning, custom heated mouse pad and ear support) and imaged from top with 5x/0.16NA dry and 25x/0.8NA immersion lenses with help of an Objective Inverter (LSM Tech). glycerol of 80 wt% (refractive index 1.44) was used as the immersion medium without any coverslip between the lens and the sample.

FLIM imaging of the ear vasculature was done as described below.

### FLIM Instrumentation, Processing and Analysis

Zeiss LSM-780 NLO confocal/multiphoton microscopy system consists of an inverted Axio Observer (Zeiss) microscope, motorized stage for automated scanning, Chameleon Vision-II (Coherent Inc.) ultrafast Ti:sapphire laser with dispersion compensation to deliver shorter pulses at the specimen plane (690–1060 nm, 80 MHz, 150 fs) for multiphoton excitation and a standard set of dry and immersion objectives. Three HPM-100 hybrid GaAsP detectors (Becker and Hickl) are connected to the non-descanned (NDD) port of the microscope using two T-adapters (Zeiss) with proper dichroics and band pass filters to collect as much fluorescence as possible in the spectral ranges Ch1: 340–380 nm (tryptophan), Ch2: 460–500 nm (NAD(P)H) and Ch3: 520–560 nm (FAD). NADH and FAD channels also contain 690 nm short pass filter (Zeiss) in the beam path. Three SPC-150 cards (Becker and Hickl) synchronized with the pulsed laser and the Zeiss LSM-780 scan head signals collect the time-resolved fluorescence in TCSPC mode using SPCM (9.74) acquisition software.

For cell monolayers a Zeiss 40 × 1.3NA oil, (EC Plan-Neuofluar, UV transmission is 60% at 340 nm) objective lens and 5x/0.16NA dry and 25x/0.8NA immersion lenses for cancer xenograft was used to focus the light on the sample and collect the emission for 60 sec. The average power at the specimen plane (7 mW) and the acquisition time was chosen to reduce any photo damage to the cells. For intravital FLIM imaging of cancer xenograft the excitation laser power was increased with imaging depth to maintain the detected photon rate around 10^5^ photons/s (see Fig. 7A).

After simultaneous acquisition of FLIM images for Trp and NAD(P)H at 740 nm excitation, immediately followed by FAD excitation at 890 nm (Supplementary Information), the fluorescence lifetime images (256 × 256 pixels) were fitted for 2-components at optimized χ^2^ (~1) using SPCImage software (v. 5.5, Becker & Hickl). A number of parameters were generated including photon images, τ_1_, τ_2_, a_1_%, a_2_%, and χ^2^ for each pixel of each channel. The acquired images by FoV were segmented using manual cell segmentation and thresholded by 2 × 2 pixels/ROI, based on NAD(P)H photon images to isolate mitochondrial morphologies^28^ and whole cell (without nucleus) ROIs; further details are provided in Supplementary Information. For the cancer xenograft model, the tumor was imaged at different depths and these different depths were selected for ROI based FLIM analysis (see Fig. 7).

The large image data sets produced from the number of data parameters for each ROI, from 3 FLIM detectors capturing 3 auto-fluorescent probes, excited at different wavelengths and emitting in different spectral ranges, were further analyzed by a modular and adjustable, method to process large FLIM image datasets, by combining ImageJ/Fiji (NIH) and Excel (Microsoft Office) custom macros and template spreadsheets.

### Quantification of the FLIM Optical Redox Ratio

For the optical redox ratio calculation, we have used a FLIM-based method (FLIRR) which uses NAD(P)H-a_2_%/FAD-a_1_% redox pairs. Our preferred FLIRR avoids potential intensity-related artefacts, such as photo-bleaching, light-scattering or fluctuations of illumination levels.

### Calculation of Trp E%

E% is calculated using the generalized equation of ***E*** **= 1** **−** **(** ***τ***_***DA/***_ ***τ***_***D)***_ where T_DA_ is the lifetime of the double-label specimen and T_D_ is the mean lifetime of the single-label specimen. The commonly accepted T_DA_ is Tm, the amplitude-weighted average lifetime, consisting of the fraction of the unquenched donor (a2%) multiplied by its lifetime plus the fraction of the quenched donor (a1%) multiplied by its lifetime (***τ ***m = (***τ***1 * a1% + ***τ***2 * a2%)). ***τ***_D_ in the case of Trp is the published single donor Trp in solution at 3.1 ns^13,31^. Consequently the calculation of E% here = (1 − (***τ***m/3.1)) * 100

### Generating color-coded images

SPCImage software facilitates export of several color-coded images with adjustable color ranges. It does not specify NADH-a_2_%/FAD-a_1_% ratio images included in this method paper; these were generated in MATLAB based on original data.

### Data availability

All data generated or analyzed in this study are included in this manuscript (and in the Supplementary Information)

## Electronic supplementary material

Supplementary information
