# Supplementary material for: Segmented cell analyses to measure redox states of autofluorescent NAD(P)H, FAD & Trp in cancer cells by FLIM
Source: Sci Rep. 2018 Jan 8;8:79. doi: 10.1038/s41598-017-18634-x (PMC5758727; doi:10.1038/s41598-017-18634-x)
Supplement: Supplementary file 1 — Supplementary information [file 41598_2017_18634_MOESM1_ESM.pdf]

**Supplementary Information**

# Segmented cell analyses to measure redox states of autofluorescent NAD(P)H, FAD & Trp in cancer cells by FLIM

**Horst Wallrabe[1,2], Zdenek Svindrych[1,2], Shagufta R. Alam[1,2], Karsten H. Siller[4], Tianxiong Wang[3], David Kashatus[5], Song Hu[3], and Ammasi Periasamy[1,2,3*]**

[1]The W.M. Keck Center for Cellular Imaging, [2] Departments of Biology, [3]Biomedical Engineering, [4] Advanced Research Computing Services, [5]Microbiology, Immunology & Cancer Biology, University of Virginia, Charlottesville, VA, USA.

## Contents

**1. Process Flow Chart Overview (details in each section below)**

**2. Specimen preparation for imaging**

**3. Imaging Protocol**

- **Setting up before imaging**
- **Imaging**

**4. Image Processing**

- **FLIM fitting with B&H SPCImage software**
- **ImageJ/Fiji plug-ins and macros**

**5. Data Analysis**

- **Excel macros and templates**

## 1. Process Flow Chart - Overview

| |
|---|
| 1. Specimens: optimized cell culture preparation on coverslips or glass-bottom dishes includes 70-80% confluence with overnight intervention application for the starving (5.5 mM glucose HBSS) or 50 µM $CoCl_2$ protocol. |
| 2. Next day: Prepare microscope setup, heated stage assembly, blood-gas supply, check 2-P laser output, measure Instrument Response Function (IRF), prepare folders for saving images. |
| 3. Microscopy (1) Control: Record & save Fields-of-View (FoV) with 740 nm laser on confocal, darken room, switch to non-descan & FLIM filters, record/save images, switch to 890nm for FAD acquisition record/save images. |
| 4. Microscopy (2) Treat: Without disturbing the specimen in the temperature controlled chamber on stage, treat the specimen with phenol-free 25 mM glucose media (final) or doxorubicin, re-image same FoVs 30 minutes later or time course. Copy .sdt images. |
| 5. FLIM-fitting .sdt images with B & H - SPCImage software: setting up conditions with measured IRF, optimizing $Chi^2$, batch processing and exporting .asc data files & .tif image files |
| 6. Create .tif photon image stack for NADH with custom ImageJ macro, which converts previously assembled folder with .asc photon images. This is used for ROI selection. |
| 7a. Load .tif photon stack in ImageJ, open custom ROI plug-in, set ROI size and thresholds to match morphology in controls and glucose challenge images; save<br>7b. Load .tif photon stack in ImageJ, manually zero nuclear region, create individual complete cell segmented outline ROIs with ROI Manager; save .zip |
| 8. Run ImageJ/Fiji custom plug-in to combine the ROIs .zip files of 7a & b, creating a new combined ROI.zip folder, allocating ROIs to individual cells for the next step |
| 9. Run ImageJ/Fiji custom macro, computing results by ROI within single, segmented cells/by FoV set based on exported .asc data files ex step 5 and .zip folders ex step 8. Results are automatically saved. |
| 10. ‘Results’ are processed in Excel custom macro to produce means, frequency distribution histograms & means bar charts for key parameters. Save additional non-formula copy |
| 11. Use the ‘non-formula’ copy of Excel workbook to conduct additional computations or transfer selective data to a new workbook other suitable analysis software. |

## 2. Specimen preparation for imaging

Based on above protocols, coverslips with cells were mounted in metal chambers, maintained under physiological conditions at 37°C on a temperature controlled microscopy stage (PeCon Tempcontrol) under humidified blood-gas flow (5% $CO_2$, 21% $O_2$, balance $N_2$).

## 3. Imaging Protocol

(FLIM microscopy instrumentation see manuscript) **Setting up before imaging**

Before mounting coverslip specimens in imaging chambers, all imaging-related elements are activated: multiphoton laser, Zeiss and Becker & Hickl computer/software, 40x 1.3 oil objective, heated stage, humidified blood-gas mix at correct flow rate, folders created for images to be saved in. Finally, the first coverslip is mounted and placed on temperature-controlled stage. Plan to measure and record instrument response function (IRF) at the completion of imaging cells.

**Imaging** FLIM imaging proceeds along the following steps

1. On the Zeiss 780, the first FoV is selected through the ocular with brightfield illumination.
2. A Zeiss 40x 1.3NA oil, (EC Plan-Neuofluar, UV transmission is 60% at 340 nm) objective lens was used to focus the light on the sample and collect the emission.
3. Switch to 740 nm/2% power (7-8 mW) excitation. The signal is NADH autofluorescence. Adjust focal plane, record position. Depending on the cell culture, the next FoV may need to be again selected through the ocular. Also possible is to continue with 740 nm, selecting the next FoV on screen. Continue to record and save positions for 5-10 FoVs.
4. Activate FLIM acquisition software including 60 s acquisition time. On the Zeiss 780, activate FoV position #1, scan at 740 nm to fine-tune focal plane, stop, switch to non-descan and the appropriate FLIM emission filter set, start scanning again and simultaneously ‘Start’ FLIM software with automatic count-down of 60 s acquisition. Save image set of the 3-channel output (Tryptophan, NADH, and FAD-channel, being mostly NADH spectral bleed-through at 740 nm illumination)
5. Without changing the non-descan mode, quickly switch wavelength to 890 nm/5% power (23 mW) for FAD acquisition, following FLIM steps in (3). Repeat steps for all recorded FoV positions.
6. Without disturbing the imaging chamber on stage, add treatment/exchange media to the desired final molarity, record the time, and return (in our case 30 min for glucose challenge or 15 min interval for doxorubicin time course) to re-image the identical FoV positions as per steps 3 & 4.
7. Measure/record instrument response function (IRF) with standard urea crystal specimen

## 4. Image Processing

### FLIM fitting with B&H SPCImage software

The saved original .sdt image files are processed with the SPCImage software (version 5.5). The B&H handbook provides extensive detail and offers many options for different fitting strategies (Becker & Hickl user manual, 7th edition, 2017). The major settings used for this protocol include: measured IRF, multi-exponential fit model, 2-component fitting (bi-exponential fit; non-linear least square fitting) for tryptophan (Trp), NADH, FAD. The prime objective is to achieve high enough photon count with a $Chi^2$ as close as possible to 1, but less than 2. The following are the major fitting process steps:

<u>NOTE</u>: Since we acquired two image sets, one at 740 nm and one at 890 nm excitation, each image set is fitted separately. The 740nm image names have a _c1_ suffix; the 890 nm set a _c2_. Each excitation has 3-channel output, although only Ch1 & 2 of 740 nm (Trp & NADH) and Ch3 of 890 nm are eventually analyzed.

1. Import the first .sdt image file. Note: 'Module' in the pop-up needs to be adjusted to the correct detector numbers – in our case 3.
2. Set up conditions as outlined above, including storing and loading measured IRF and channel-specific settings.
3. To establish an acceptable $Chi^2$ value: unclick 'Shift', pass cursor over several pixel locations of morphology of interest for target $Chi^2$, fix 'Shift' at that choice. Repeat for all channels.
4. 'Calculate' decay matrix 'All channels', followed by selecting the batch processing procedure for the whole image set (all FoVs).
5. Batch export all data, producing .asc data files and some color-coded .img tiff files.

### ImageJ/Fiji plug-ins and macros

The substantial data output from SPCImage is managed by custom plugins and macros, including the important, semi-automated ROI selection process, which currently relies partly on the scientist's judgement on which thresholds best achieve ROI placement on target morphology. As per flow chart, there are several sequential steps:

1. For ROI selection, the NADH photon images […. _c1_-Ch2-_photons.asc], showing mitochondrial and other cellular features, need to be converted into .tif stacks. Our ImageJ or Fiji plugin ->custom macros - achieves that

2. Open the Control photon stack in ImageJ. Custom plugins ROI selection popup (see insert). For discrete placement of ROIs we choose a 2x2 pixel ROI. The lower threshold of this 12-bit example is key and must be fine-tuned, until ROI placement on target morphology is satisfactory. As the plugin will process the stack at that setting, this may not work for all images in the stack. In this case the stack is processed at different thresholds, saved and later a new ROI folder is created, merging .zip files with their correct individual thresholds. The alternative is to process individual images instead of a stack. This careful process is particularly important, when controls and identical FoV treatment images have different intensities, even though they are showing the same cellular features[10,11]. For cell segmentation, the photon stack is again opened, the ImageJ/Fiji 'ROI manager' feature used to select the individual cell shapes manually (see Fig. 1-B, Manuscript) and save as .zip file.

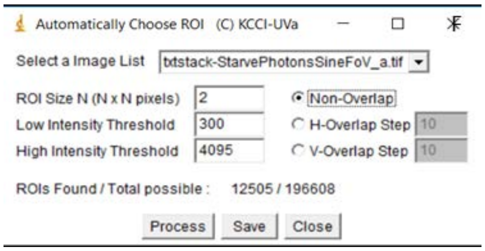


3. If the target is to cover the whole cell with ROIs, a lower threshold in the 'Automatically choose ROI' plugin will achieve that. If the nucleus is to be excluded, fine-tuning the threshold may also achieve that, provided the nuclear region is sufficiently different in intensity; alternatively, the nuclear region can be 'zeroed' easily in ImageJ/Fiji by the 'free-hand selection' tool -> Edit -> Clear, after which the plugin is applied.

4. The two .zip folders of step 2 & 3 further processed with the custom plugin to allocate the ROI selection in step 2 to the confines of cell shapes created in step 3. To compute all FLIM data output from SPCImage by ROIs/by cell and FoV, another custom macro automates the process produces text file results for parameters displayed as shown below. In the next Data Analysis phase, ratios and correlations are generated, based on these results.

| K | L | M | N | O | P | Q |
|---|---|---|---|---|---|---|
| trp t1 | trp a1 | trp photons | trp chi2 | trp shift | trp scatter | trp offset |

| R | S | T | U | V | W | X | Y | Z |
|---|---|---|---|---|---|---|---|---|
| FAD t1 | FAD t2 | FAD a1 | FAD a2 | FAD photons | FAD chi2 | FAD shift | FAD scatter | FAD offset |

| | A | B | C | D | E | F | G | H | I | J |
|---|---|---|---|---|---|---|---|---|---|---|
| 1 | ROI # | NADH t1 | NADH t2 | NADH a1 | NADH a2 | NADH ph | NADH chi | NADH shift | NADH scatter | NADH offset |

## 5. Data Analysis

### Analyzing results with Excel macros and templates

Based on the large data pool, the analysis opportunities are substantial; particularly when including ratios, frequency distributions, correlation scatter charts and means bar charts. With the ability of FLIM microscopy – based on lifetime - to determine fractions of bound populations (a1 for Trp, a2 for NADH and a1 for FAD), exploring biological questions are made possible. Anyone familiar with Excel functions can extract the data for further computations or tailor a macro to their own needs.
